# Dietary pattern and telomere length in preschool children in a middle‐income country

**DOI:** 10.1111/mcn.13146

**Published:** 2021-02-04

**Authors:** Seyed Elyas Meshkani, Akram Kooshki, Ahmad Alahabadi, Moslem Lari Najafi, Abolfazl Rad, Forough Riahimanesh, Mohammad Miri

**Affiliations:** ^1^ Cellular and Molecular Research Center Sabzevar University of Medical Sciences Sabzevar Iran; ^2^ Department of Nutrition & Biochemistry, School of Medicine Sabzevar University of Medical Sciences Sabzevar Iran; ^3^ Non‐Communicable Disease Research Center, Department of Environmental Health Sabzevar University of Medical Sciences Sabzevar Iran; ^4^ Pharmaceutical Sciences and Cosmetic Products Research Center Kerman University of Medical Sciences Kerman Iran

**Keywords:** ageing, food groups, preschool children, telomere length

## Abstract

Telomere length (TL) has been associated with lifestyle and dietary pattern. However, the available evidence on this association in children is scarce, especially in low‐ and middle‐income countries (LMICs). Therefore, this study aimed to evaluate the association of dietary pattern and leukocyte TL (LTL) in preschool children, Sabzevar, Iran (2017). This cross‐sectional study was based on 187 preschool children (aged 5 to 7) recruited from 27 kindergartens. Nutrition information including amounts of consumed dairy products, meat and processed meat products, nuts and seeds, white bread and refined grains, fruits, vegetables, simple sugars, fats and drinks was obtained through a questionnaire. Linear mixed‐effects models were fitted with polymerase chain reaction (PCR) plate ID and kindergartens as random effects to estimate the association of each food group consumption with LTL, controlled for relevant covariates. Higher consumption of dairy products and sugar was associated with shorter LTL (*β* = −0.180, 95% confidence interval [CI]: −0.276, −0.085, *P* value <0.001 and *β* = −0.139, 95% CI: −0.193, −0.086, *P* value <0.001, respectively). An increase in consumption of fish, nuts and seeds, coloured fruits, green leafy vegetables, cruciferous vegetables and olive was significantly associated with the increase in relative LTL. The associations for the consumption of legumes, other fruits, yellow and orange vegetables, red meat, egg, white bread and refined grains, solid and liquid fats, processed meats, potato chips, carbonated drinks, tea (black) and soft drinks groups were not statistically significant. Our findings showed that there was an association between the consumption of certain food groups with LTL.

**Abbreviations**
CI: confidence interval
LTL: leukocyte telomere length
qRT‐PCR: quantitative real‐time polymerase chain reaction
TL: telomere length

**Key messages**

- The association of dietary pattern and leucocyte telomere length was investigated in preschool children of Sabzevar, Iran.
- Higher consumptions of dairy products and sugar were associated with shorter LTL.
- An increase in consumption of fish, nuts and seeds, colored fruits, green leafy vegetables, cruciferous vegetables, and olive were significantly associated with the increase in relative LTL.
- The associations for the consumption of legumes, other fruits, yellow and orange vegetables, red meat, egg, white bread and refined grains, solid and liquid fats, processed meats, potato chips, carbonated drinks, tea (black) and softdrinks groups were not statistically significant.

## 1 INTRODUCTION

Chromosomes' ends are insulated by a unique structure called telomere, consisting of structural shelterin proteins and a specific sequence (TTAGGG) of DNA (De Lange, 2005). The telomeres are responsible for protecting genetic material from deterioration and adhesion of chromosomes to each other (Lu et al., 2013). After every cell division, the length of telomere is shortened due to the hayflick limit. When shortening reaches a critical extent, it jeopardizes cell viability by affecting encoding genes, and this is the state of cellular senescence. The telomere length (TL) is mainly affected by inheritance, but environmental factors are of importance (Cassidy et al., 2010). It has been previously shown that oxidative stress and inflammation impact the leukocyte TL (LTL). Of note, lifestyle is another important factor that determines the attrition of the telomere (De Meyer et al., 2011; Shammas, 2011). According to above, LTL reflects the influence of the environment on the genetic matter, which in turn determines pace of the ageing process (Hornsby, 2007) and telomere‐related diseases such as cancer (Shay & Wright, 2011), cardiovascular diseases (Serrano & Andrés, 2004) and diabetes (Salpea & Humphries, 2010).

Epidemiological studies have successfully established an excellent link between LTL, inflammation, oxidative stress and the ageing process (Paul, 2011; Price et al., 2013). These factors contribute to an accelerated TL shortening, which brings various diseases related to ageing in populations (Cassidy et al., 2010). However, to date, a clear mechanism that describes the causality between ageing and TL has not been established yet. Nonetheless, studies have suggested that lifestyle‐related activities such as smoking (Müezzinler et al., 2015), exercise (Nomikos et al., 2018) and diet (Paul, 2011) are playing a role in the TL.

Diet, as a part of the lifestyle, was associated with TL (García‐Calzón et al., 2015). Earlier studies have reported that body mass index (BMI) is affected by diet/nutrition (Huang et al., 2005; Kooshki & Golafrooz, 2009; Shirzadeh et al., 2016). In parallel, other reports have suggested that BMI also affects TL (Valdes et al., 2005), which brings the role of diet on TL and its related diseases to importance. Mainly nutrition status poses its effect on the TL by three main routes, first by regulating TL, second by regulating the activity and function of telomerase enzyme and third by inhibiting DNA damage and oxidative stress (Paul, 2011). Micronutrients such as vitamins, minerals and bioactive foods are involved in the suppression of inflammatory response and oxidative stress, which lead to reduced DNA damage and hence longer TL (Paul, 2011; Shen et al., 2009).

An emerging body of evidence reported an association between dietary patterns and food groups with TL (Freitas‐Simoes et al., 2016; Kiefer et al., 2008; Nettleton et al., 2008; Paul, 2011); however, the available evidence on the dietary pattern and LTL is scarce yet (García‐Calzón et al., 2015). Moreover, there is no study on this association in low‐ and middle‐income countries (LMICs). Therefore, this study aimed to investigate the association between the general nutrition pattern of preschool children and their LTL.

## 2 MATERIAL AND METHODS

### 2.1 Study area

This study was carried out in Sabzevar, a city located in Razavi Khorasan Province, north‐east of Iran. The coordination is as follows: 36°12′N, 57°35′, elevation: 977 m. Sabzevar has an arid climate. The population of this city is approximately 240,000 based on the last census in 2016, in which the number of men and women is almost equal.

### 2.2 Study population

**FIGURE 1:**
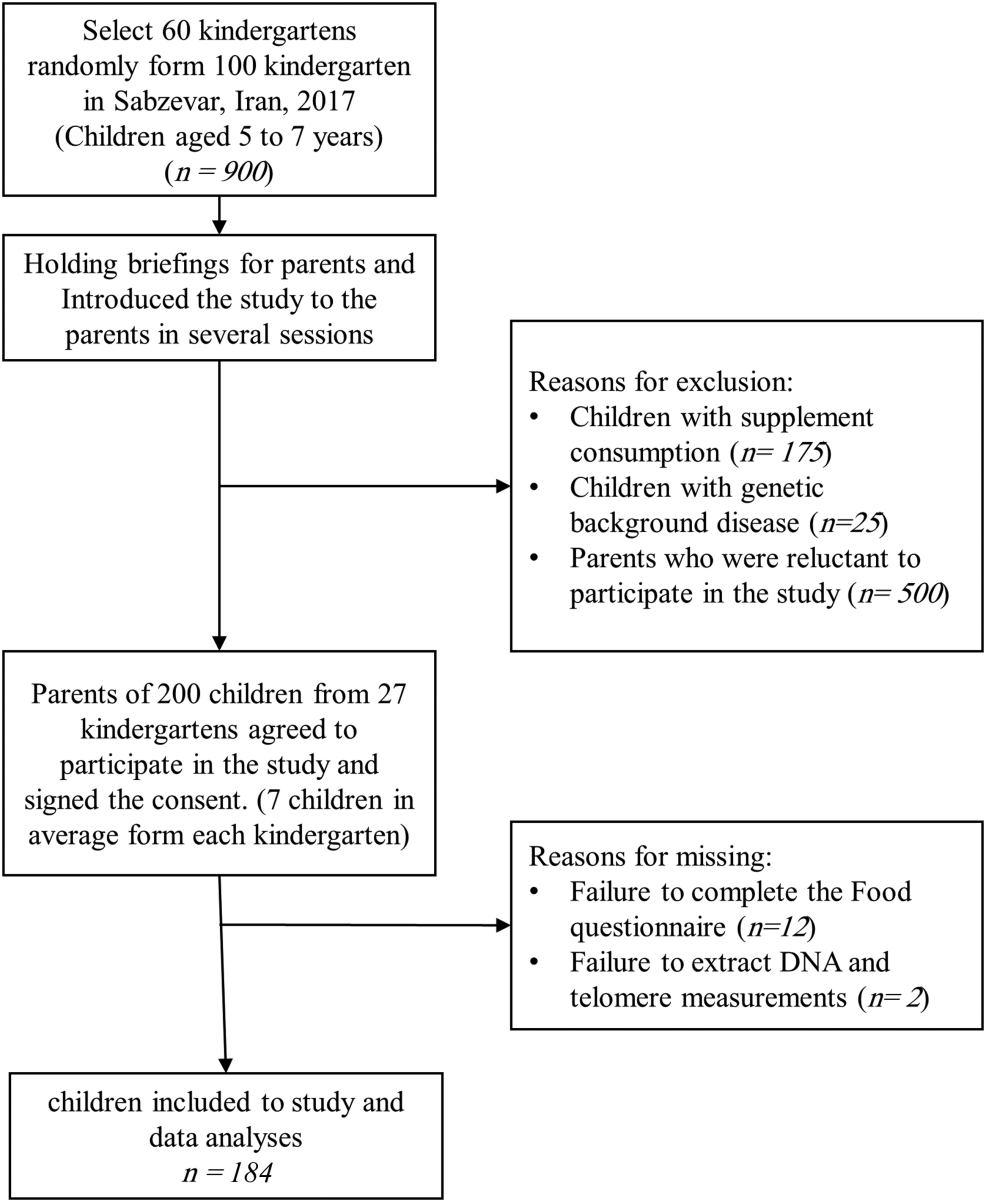
Flow chart for the inclusion of participants in the study

This cross‐sectional study was performed in 2017 in preschool children aged 5 to 7 years. From about 100 kindergartens in Sabzevar, 60 of them were randomly selected for this study. The study was introduced to the parents in several sessions. Finally, 184 children from different families and 27 kindergartens (seven children in average from each kindergarten) agreed to enrol their children in the study. The participants were healthy children without any specific supplement consumption and no genetic background disease. Gender, age, weight, maternal and paternal education status, economic status and exposure to environmental tobacco smoke were debriefed by questionnaires completed by parents (Figure 1).

### 2.3 Nutrition information

The questionnaire to collect the required nutritional information was completed by parents and has been previously validated by Esfahani et al. (2010). Information about the consumption included dairy products (e.g. milk, cheese, yogourt and curd), red meat, fish (fish or tuna), nuts and seeds (e.g. pistachios, almond, sesame, hazelnut and peanut), egg, legumes (e.g. chickpea, lentil and kidney bean), white bread and refined grains (e.g. rice and pasta), coloured fruits, other fruits (e.g. apple, banana and kiwi), yellow and orange vegetables (e.g. carrot, zucchini and tomatoes), cruciferous vegetables (e.g. cabbage and cauliflower), green leafy vegetables (e.g. mint, parsley and spinach), simple sugar (e.g. cube sugar and candy), solid and liquid fats (e.g. hydrogenated oil, mayonnaise and animal fats), processed meats (e.g. sausages and ham), potato chips, carbonated drinks (e.g. soda and nonalcoholic beer), tea (black), soft drinks (e.g. sweetened fruit juices) and olive (olive and olive oil). The quantity of consumption of mentioned foods groups was captured using an ordinal response variable with the levels in two overall categories, that is, never or once per month or once per week or two or three times per week (as reference) and consumption once per day or two or three times per day or per each meal (respective to the type of food, i.e. grams and litre).

### 2.4 LTL measurement

Two millilitres of blood was taken from each child in the morning at the reference lab of Sabzevar and transferred to laboratory in Vacutainer® Plus Plastic K2EDTA Tubes (USA) within less than 2 h and kept in −80°C until analyses. The DNA was extracted from blood samples using DNA extraction kit (GeNet Bio, Korea) according to the manufacturer's instructions (http://www.genetbio.com/en/page5.html). The purity ratio (A260/230) and yield (A260/280) (ng μl^−1^) of extracted DNA samples were determined using Nanodrop spectrophotometer (BIO INTELLECTICA Nano100, Canada). All DNA samples were stored at −80°C until the time of analyses.

To measure LTL, we performed quantitative real‐time polymerase chain reaction (qRT‐PCR) based on the method by Cawthon, which determines telomere copy number relative to single‐gene (human beta globin) copy number (*T*/*S* ratio) (Cawthon, 2002). This ratio is known as relative LTL. qRT‐PCR was carried out using SYBR Green PCR Master Mix 2× (Amplicon, Denmark) on a CFX96 Touch™ Real‐Time PCR Detection System (Bio‐Rad, USA) with the following primers: telomere forward 5′‐CGGTTTGTTTGGGTTTGGGTTTGGGTTTGGGTTTGGGTT‐3′, telomere reverse 5′‐GGCTTGCCTTACCCTTACCCTTACCCTTACCCTTACCCT‐3′, single‐copy gene forward 5′‐GCTTCTGACACAACTGTGTTCACTAGC‐3′ and single‐copy gene reverse 5′‐CACCAACTTCATCCACGTTCACC‐3′. Telomere and single‐copy numbers were evaluated in triplicate (three measured per individual) in each qPCR reactions. A total of 10 plates were processed. Each reaction for telomere evaluation contained 5 μl of SYBR Green PCR Master Mix 2×, 0.1 μl of forward primer (10 pmol μl^−1^), 0.9 μl of reverse primer (10 pmol μl^−1^) and 1 μl (25 ng μl^−1^) of DNA. Single‐copy gene evaluation was performed as the telomere reaction, with the exception that we added 0.3 μl (10 pmol μl^−1^) of forward primer and 0.7 μl (10 pmol μl^−1^) of reverse primer. The cycling thermal profile was the same for telomere and single‐copy gene, including one cycle at 95°C for 15 min, followed by 50 cycles of 95°C for 15 s, 60°C for 20 s and 72°C for 20 s. Each plate contained five serial concentrations of a standard human genomic DNA sample (150, 50, 16.7, 5.55 and 1.85 ng per well), giving amplification efficiencies ranging from 95% to 105%. Melting curve analyses were used at the end of each cycle to confirm amplification specificity and absence of primer dimers. Finally, the relative *T*/*S* ratio was calculated as follows (Cawthon, 2002; Cawthon, 2009):

(1) $$\text{Relative}T/S=\frac{2^{-\left({\mathrm{Ct}}_{\mathrm{TL}}-{\mathrm{Ct}}_{\mathrm{SCG}}\right)\text{sample}}}{2^{-\left({\mathrm{Ct}}_{\mathrm{TL}}-{\mathrm{Ct}}_{\mathrm{SCG}}\right)\text{control}}},$$

where Ct_TL_and Ct_SCG_ were the average Ct of triplicate for each sample and single‐copy gene. In this study, the average value of *T*/*S* ratio of samples (the numerator) was used as control (the denominator).

To provide reproducibility, interassay variability was measured in three different days (% of coefficient of variation [%CV] was 0.74% for LTL and 47% for single‐copy gene).

### 2.5 Statistical analysis

#### 2.5.1 Main analysis

Considering the multilevel nature of our data, we fitted linear mixed‐effects models with LTL as the outcome, indicators of nutrition (one at a time) as fixed effect predictor and PCR plate ID and kindergartens as random effects (one at a time). We also adjusted our models for a priority of selected variables: age (continuous), gender (boy/girl), BMI (continuous) and two indexes of household socio‐economic status (SES), also neighbourhood SES. The paternal and maternal education level (uneducated/primary education, middle school education or university education) and income (≥15 million, 15 to 30 million and ≤30 million rials) were considered as household SES. Neighbourhood SES was defined by the census conducted in 2016, based on the per cent of illiteracy and unemployment of adults in each census tract. Coefficiencies were defined by the number of meals with contracted scores (never or once per month, once per week and two or three times per week = 1, once per day and two or three times per day = 2). A significance level of 0.05 was considered for the whole study. All statistical analyses were performed in StataMP Version 15 (StataCorp LP, College Station, TX).

#### 2.5.2 Sensitivity analyses

The models were further adjusted for age squared (continues), exposure to environmental tobacco smoke (yes/no), car ownership (yes/no) and home ownership (yes/no).

### 2.6 Ethical considerations

All procedures were held under the supervision of the Clinical Research Ethical Committee of the Shahid Sadoughi University of Medical Sciences, Yazd, Iran (IR.SSU.SPH.REC.1395.66), and approvements were signed by parents.

## 3 RESULTS

### 3.1 Study population

A summarized description of the sociodemographic information of the participants is presented in Table 1. Of 184 children enrolled in this study, 106 (57.6%) were girls and 78 (42.4%) were boys. The mean (SD) age of cases was 6.4 (0.78). Both fathers and mothers' education level was mostly high school education (56.5% and 48.4%, respectively). Exposure to tobacco smoke at home was low (13%). Most of the children's families had a low income (lower than 1.5 million rials per month, 87.5%). The median of relative TL (*T*/*S* ratio) in girls was higher than boys (0.881 vs. 0.784), but this difference did not reach statistical significance.

**TABLE 1:** Descriptive statistics of demographic information, socio‐economic status and TL of subjects

Variables	*N* ^a^ (missed)	In study year
Age (years), mean ± SD	183 (1)	6.40 ± 0.78
Sex	184	
Female, *N* (%)		106 (57.60)
Male, *N* (%)		78 (42.40)
BMI (kg m^−2^), mean ± SD	168 (16)	15.26 ± 3.26
**Paternal education**	184	
No education/primary school, *N* (%)		64 (34.80)
Secondary school education, *N* (%)		104 (56.50)
University degree or higher, *N* (%)		16 (8.70)
**Maternal education**	184	
No education/primary school, *N* (%)		71 (38.60)
Secondary school education, *N* (%)		89 (48.40)
University degree or higher, *N* (%)		24 (13)
**Income**	181 (3)	
≥15 million rials, *N* (%)		161 (86.50)
15 to 30 million rials, *N* (%)		19 (10.30)
≤30 million rials, *N* (%)		1 (0.50)
Tobacco exposure at home, *N* (%)	183 (1)	24 (13)
Illiterate per cent per census tract (%), median (first Q to the third Q)	184	25.67 (7.00–35.01)
Unemployed per cent per census tract (%), median (first Q to third Q)	184	7.00 (2.98–10.78)
**Relative telomere length (*T*/*S* ratio), median (first Q to the third Q)**	184	
Overall		0.84 (0.52–1.23)
Female		0.88 (0.54–1.25)
Male		0.78 (0.47–1.16)

*Note:* Data are presented as mean ± SD, *N* (%) and median (first Q to the third Q).

Abbreviations: BMI, body mass index; Q, quartile; SD, standard deviation.

^a^ Number of available observations.

Based on the given answers to questionnaires, we found that most of the participants had a high intake of white bread and refined cereals (88.6% two or three times per day), which is due to the consumption of white rice as a major food source in Iranian people. Consumption of Brassicaceae was relatively low (52.7% never or once per month). Fish consumption in our subjects was also low in our study population (56.5% never or once per month). Intake of processed meats and olives was also relatively low in our subject population (87% and 72.3% never or once per month, respectively). Our data showed that dairy intake was ordinary 29.9% of subjects had one meal per day at least, whereas sugar had a higher intake rate, 22.3% once per day and 42.4 two or three times per day. The amounts of consumed food groups are thoroughly presented in Table S1. The size of portions was measured by scales described elsewhere (Esfahani et al., 2010).

Furthermore, to evaluate the correlation between consumption of food groups, we conducted the Spearman correlation test (Table S2). We found a significant correlation between consumption of coloured fruits with fish (0.21), nuts and seeds (0.24), legumes (0.19), and, more importantly, with yellow and orange vegetables (0.31). These correlations point towards a healthy lifestyle among members of the study population. Also, our data demonstrated a considerable correlation between intake of sugar and oil (0.47), same as to cruciferous vegetables and fish (0.48), green leafy vegetables (0.43) and also olive (0.40). Besides, we found a strong correlation between olive and fish consumption (0.42).

### 3.2 Main analysis

The estimated associations between nutrition status and relative LTL are presented in Table 2. In the fully adjusted model, an increase in the consumption of dairy products and simple sugar was associated with shorter LTL (*β* = −0.180, 95% confidence interval [CI]: −0.276, −0.085, *P* value <0.001 and *β* = −0.139, 95% CI: −0.193, −0.086, *P* value <0.001, respectively). There was a significant positive association between the consumption of fish and relative LTL in children (*β* = 0.208, 95% CI: 0.144, 0.272). Our analyses indicated that increased consumption of nuts and seeds, coloured fruits, green leafy vegetables, cruciferous vegetables and olive was significantly associated with the increase in relative LTL (Table 2). Although the consumption of legumes, other fruits, yellow and orange vegetables had a positive association with relative LTL, these associations were not statistically significant. Moreover, meats, egg, white bread, refined grains, oil, processed meats, potato chips, carbonated drinks, tea (black) and soft drinks groups demonstrated an inverse relationship with relative LTL. However, these associations were not statistically significant (Table 2).

**TABLE 2:** Linear mixed‐effects model for the consumption of foods and consequent effects on TL of subjects

Food groups		*β* coefficient (95% CI)	*P* value
Dairy products	Model 1	−0.198 (−0.276, −0.120)	<0.001
	Model 2	−0.180 (−0.276, −0.085)	<0.001
Red meat	Model 1	−0.060 (−0.134, 0.012)	0.103
	Model 2	−0.044 (−0.130, 0.421)	0.314
Fish	Model 1	0.215 (0.161, 0.270)	0.000
	Model 2	0.208 (0.144, 0.272)	0.001
Nuts and seeds	Model 1	0.130 (0.075, 0.185)	<0.001
	Model 2	0.105 (0.041, 0.168)	0.001
Egg	Model 1	−0.006 (−0.098, 0.086)	0.898
	Model 2	0.008 (−0.111, 0.127)	0.894
Legumes	Model 1	0.198 (−0.058, 0.097)	0.626
	Model 2	0.004 (−0.086, 0.097)	0.930
White bread and refined grains	Model 1	−0.128 (−0.274, 0.017)	0.083
	Model 2	−0.958 (−0.249, 0.057)	0.219
Coloured fruits	Model 1	0.092 (0.030, 0.155)	0.004
	Model 2	0.115 (0.047, 0.183)	0.001
Other fruits	Model 1	0.062 (0.010, 0.114)	0.190
	Model 2	0.076 (0.019, 0.134)	0.009
Yellow and orange vegetables	Model 1	0.080 (0.019, 0.140)	0.010
	Model 2	0.093 (0.020, 0.166)	0.012
Green leafy vegetables	Model 1	0.090 (0.036, 0.143)	0.001
	Model 2	0.098 (0.037, 0.159)	0.002
Cruciferous vegetables	Model 1	0.128 (0.071, 0.179)	<0.001
	Model 2	0.126 (0.067, 0.184)	<0.001
Simple sugar	Model 1	−0.132 (−0.178, −0.086)	<0.001
	Model 2	−0.139 (−0.193, −0.086)	<0.001
Solid and liquid fats	Model 1	−0.047 (−0.104, 0.010)	0.110
	Model 2	−0.040 (−0.105, .024)	0.219
Processed meats	Model 1	−0.047 (−0.146, 0.054)	0.366
	Model 2	−0.033 (−0.156, 0.088)	0.585
Potato chips	Model 1	−0.032 (−0.094, 0.030)	0.310
	Model 2	−0.055 (−0.126, 0.014)	0.121
Carbonated drinks	Model 1	−0.012 (−0.070, 0.045)	0.677
	Model 2	−0.027 (−0.098, 0.042)	0.435
Tea (black)	Model 1	−0.020 (−0.068, 0.028)	0.416
	Model 2	−0.012 (−0.071, 0.045)	0.667
Soft drinks	Model 1	−0.009 (0.063, 0.044)	0.723
	Model 2	0.017 (−0.044, 0.080)	0.573
Olive	Model 1	0.172 (0.120, 0.224)	<0.001
	Model 2	0.165 (0.108, 0.224)	<0.001

*Note:* Model 1: crude (not adjusted) model. Model 2: this model is adjusted for age, sex, body mass index, paternal and maternal education level, income, tobacco smoke exposure at home, illiterate per cent per census tract and unemployed per cent per census tract. Polymerase chain reaction plates and kindergarten were considered as a random effect in all analyses.

Abbreviations: CI, confidence interval; TL, telomere length.

### 3.3 Sensitivity analyses

Furthermore, the results of the models further adjusted for age squared, exposure to environmental tobacco smoke, car ownership and home ownership were generally similar to the main analyses in terms of statistical significance and direction (data not shown).

## 4 DISCUSSION

To the best of our knowledge, this is the first report of a relationship between various nutrient groups and LTL on preschool children in LMICs. This study was reinforced by detailed information on types of food and number of meals in a specific period of time. Our study showed that LTL was positively associated with the consumption of fish, all types of nuts and seeds, coloured fruits, olive, cruciferous vegetables, and green and orange vegetables. Moreover, our analysis demonstrated that increase in intake of dairy products and simple sugar was associated with shorter LTL. The consumption of other food groups such as red meat, egg, white bread and refined grains, solid and liquid fats, processed meats, potato chips, carbonated drinks, tea (black), soft drinks, legumes, rest of fruits, and yellow and orange vegetables had no significant association with relative LTL in preschool children.

Spearman correlation showed that there was a significant correlation between consumption of some food groups, which indicated the state of leading a healthy lifestyle; for example, coloured fruits, fish, legumes, and yellow and orange vegetables had a significant correlation that also shows features of Mediterranean diet pattern (Dontas et al., 2007).

Our finding is in line with the results of previous reports in other age groups (Freitas‐Simoes et al., 2016). De Meyer et al. (2018) showed that consumption of fried potato has a direct effect on the amounts of C‐reactive protein and uric acid, indicators of oxidative stress and inflammation. Also, they linked this increase of inflammatory factors to shorter LTL in 2500 middle‐aged adults (De Meyer et al., 2018). Another study on a multiethnic group of 540 people (aged between 57.6 and 65.3 years old) showed that processed meat was strongly associated with shorter LTL. It was demonstrated that increment in the number of processed meat servings reduces *T*/*S* ratio by 0.005 per meal (Nettleton et al., 2008). A study by García‐Calzón et al. (2015) reported a negative association between white bread and cereal intake in children aged approximately 11.5 years with relative LTL. Also, they found a positive association between food with higher total antioxidant activity (TAC) and longer LTL (García‐Calzón et al., 2015). Another study reported a positive association between the consumption of cereal fibre and longer LTL in middle‐aged women. No significant association between total fat intake and LTL was observed; however, polyunsaturated fatty acid and, particularly, linoleic acid had a positive relationship with longer relative TL (Cassidy et al., 2010).

Oxidative stress as an environmental factor that originated from lifestyle, diet pattern as a part of lifestyle, tobacco smoking and alcohol consumption is one of the major causes of health problems (Al‐Gubory, 2014). Oxidative stress currently is considered as an important factor of neurodegenerative diseases (Uttara et al., 2009), cardiovascular diseases and cancer through reactive oxygen species (ROS) formation axis and causing DNA damage (Liguori et al., 2018). The telomere is a G‐rich sequence (TTAGGG) and hence is a sustainable target to accept DNA damage by ROS. ROS formation consequent of oxidative stress shapes 8‐oxo‐2′‐deoxyguanosine (8‐oxodG) within the cells, which cause DNA double‐strand breaks at GGG of telomere sequence. By this mechanism, oxidative stress is a key factor in telomere erosion (Kawanishi & Oikawa, 2004). Diet patterns a dual role, that is, regiments with high amounts of processed meats (Nettleton et al., 2008), carbohydrates, animal products and high‐fat content are considered as a cause of oxidative stress and development of related disease, and consumption of these food groups has shown a negative association with TL (Tan et al., 2018), which is also indicated by our results in preschool children. Dietary fats, which are abundant in animal products, increase the leptin level in the body, which consequently leads to inflammatory response and activation of immune cells. At this point, higher activity of immune cells ends in an imbalance between the production of ROS and cellular antioxidants such as superoxide dismutase (SOD) (Togo et al., 2018). The consumption of fruit vegetables leads to lower oxidative stress at cellular resolution and longer TL, as our data showed, that is due to their high content of vitamins, in particular, vitamins C and E (Ryan et al., 2010), which serve as an antioxidant agent (Baldrick et al., 2012). From another perspective, food groups such as fruits and vegetables, due to their low calorie, are involved in reducing oxidative stress. Low‐calorie intake results in activation of AMPK and SIRT1 cellular pathways, which contributes to an increased number of mitochondria in cells and consequently elevates the amount of cellular antioxidant enzymes like SOD (Manzanero et al., 2011), which could explain our findings on the relationship of consumption of these food groups and TL of preschool children.

To date, extensive efforts have been made to clarify and evaluate the capacity of foods in amelioration of inflammation and oxidative stress. In this way, studies have shed light on the antioxidant content of green and orange vegetables, green leafy vegetables, coloured fruits and also olive (Lobo et al., 2010), which explains the probable underlying mechanism of how olive consumption leads to the longer TL. Mediterranean diet in which seafood, vegetables and fruits are inseparable parts (Liguori et al., 2018) has shown a direct association with TL and delayed ageing process (Rafie et al., 2017). The fact that seafood is a sustainable source of antioxidants (Boccardi et al., 2016) describes the association we found between fish consumption and TL, but further studies demand to investigate oxidative stress markers simultaneously to describe the underlying mechanism. Similar to what happens in diabetes mellitus (Tabit et al., 2010), excessive intake of sugar and sweetened beverages leads to higher blood glucose, which initiates oxidative stress in endothelial cells and consequently develops into cardiovascular diseases (Prasad & Dhar, 2014). Besides, accelerated telomere shortening has been observed due to high sugar intake (Leung et al., 2014), which is in line with our findings in preschool children. Also, the consumption of nuts and seed has been shown to be correlated with longer telomere, and this relation was attributed to the capacity of these food groups in the reduction of oxidative stress (Tucker, 2017), which also concurs with our findings. There is an open debate about the incidence of cardiovascular diseases and the consumption of dairy. It has been suggested that the high‐fat nature of dairy products by increasing blood cholesterol and low‐density lipoprotein are suspects of these negative effects (Givens, 2017). In contrast, other researchers have shown that there is no significant association (Lordan et al., 2018). However, our data showed a negative effect of dairies on TL, which may be due to the quality and containment of milk products in Iran, mainly low‐protein content (Azizkhani & Tooryan, 2017; Mansouri‐najand et al., 2014). Whole grains are a suitable source of antioxidant (Miller et al., 2000), but it should be considered that white bread and refined grains are associated with shorter TL because of their high calorie and low amounts of beneficial nutrients (García‐Calzón et al., 2015; Nettleton et al., 2008).

The effect of socio‐economic factors and education has been investigated thoroughly before (Adler et al., 2013; Yen & Lung, 2012). In summary, better income is associated with a better quality of life, which may reduce the stresses induced by routine lifestyle; hence, the direct relationship between high income and longer TL seems logical (Yen & Lung, 2012). From the perspective of education, it seems that families with higher education status are more conscious about the nutrition facts and tend to lead a healthy lifestyle and often have a better SES (Adler et al., 2013). In line with this, we observed in families with higher education status food groups such as fish, yellow and green vegetables, green leafy vegetables and olive had a stronger effect on the TL of children.

### 4.1 Limitations

Our study had some limitations that should be considered in future studies. The sample size in this study was relatively small, which may decrease accurate results. In this study, we did not evaluate markers of oxidative stress and inflammation in preschool children to ascertain the relationship between food groups and TL. Furthermore, the quality and brand of products were not investigated, which would affect the obtained data. Importantly, we did not include calorie consumption of children in our study, which is an important agent in determining TL and oxidative stress. Moreover, we did not evaluate our subjects' genetics to adjust the effect of food groups on their TL. Physical activity and parental age were also not assessed, which could affect our results. Furthermore, the CV for the SCG assay was high (47%) and could be an additional limitation in relative *T*/*S* ratio measurements.

## 5 CONCLUSION

In conclusion, our findings showed that higher consumptions of fish, olive, green leafy vegetables, coloured fruits and cruciferous vegetables were associated with longer relative LTL in preschool children. Higher consumption of sugars (simple and soft drinks with high amounts of sugar) was associated with shorter LTL. The quality of milk and its derivatives should be considered by parents to assure the health benefits of these products. Overall, foods with higher antioxidants are important factors in preschool children's health, and parents should adopt the taste of their children to a healthy diet pattern. Our findings, if confirmed with future study, could be helpful for decision makers to provide a healthy diet programme for children in LMICs. Further longitudinal studies in other population settings are recommended.

## CONFLICTS OF INTEREST

The authors confirm that there are no conflicts of interest to declare.

## CONTRIBUTIONS

AK, SEM, FR and MM created the questionnaire, were responsible for the conception and design of the study, analysed and interpreted the data and were the major contributors in the writing of the manuscript. Telomere length was measured by AR, AA and SEM. All authors contributed to the final form of the manuscript.

## Supporting information


**Table S1.** The frequency of food groups consumption based on given answers to questionnaires
**Table S2.** Correlation test between different food groups

## Data Availability

The data that support the findings of this study are available from the corresponding author upon reasonable request.
