# Supplementary material for: Dietary pattern and telomere length in preschool children in a middle‐income country
Source: Matern Child Nutr. 2021 Feb 4;17(3):e13146. doi: 10.1111/mcn.13146 (PMC8189250; doi:10.1111/mcn.13146)
Supplement: Supplementary file 1 — Table S1. The frequency of food groups consumption based on given answers to questionnaires Table S2. Correlation test between different food groups [file MCN-17-e13146-s001.docx]

**Supplemental materials**

**Dietary Pattern and Telomere Length in Preschool Children in A Middle-Income Country**

**Table S1 Page 2**

**Table S2 Page 3**

**Table *S1*. The frequency of food groups consumption based on given answers to questionnaires**

| **Food groups** | Never or once per month, N (%) | Once per week, N (%) | Two or three times per week, N (%) | Once per day, N (%) | Two or three times per day, N (%) | Portion size |
| --- | --- | --- | --- | --- | --- | --- |
| Dairy products | 7 (3.8) | 32 (17.4) | 90 (48.9) | 55 (29.9) | 0 (0) | Cup/Tablespoon |
| Red meat | 18 (9.8) | 49 (26.6) | 87 (47.3) | 26 (14.1) | 3 (1.6) | Slice/Piece |
| Fish | 104 (56.5) | 42 (22.8) | 15 (8.2) | 21 (11.4) | 1 (0.5) | Slice |
| Nuts and seeds | 65 (35.3) | 58 (31.5) | 30 (16.3) | 23 (12.5) | 7 (3.8) | Number |
| Egg | 6 (3.3) | 32 (17.4) | 120 (65.2) | 5 (2.7) | 5 (2.7) | Number |
| Legumes | 9 (4.9) | 54 (29.3) | 96 (52.2) | 16 (8.7) | 8 (4.3) | Tablespoon |
| White Bread and refined grains | 1 (0.5) | 0 (0) | 2 (1.1) | 17 (9.2) | 163 (88.6) | Slice/Cup |
| Colored fruits | 16 (8.7) | 34 (18.5) | 84 (45.7) | 32 (17.4) | 17 (9.2) | Number/Slice |
| Other fruits | 20 (10.9) | 37 (20.1) | 54 (29.3) | 41 (22.3) | 31 (16.8) | Number/Slice |
| Yellow and orange vegetables | 21 (11.4) | 54 (29.3) | 55 (29.9) | 43 (23.4) | 10 (5.4) | Cup/Number/Tablespoom |
| Green leafy vegetables | 62 (33.7) | 47 (25.5) | 35 (19.0) | 32 (17.4) | 7 (3.8) | Cup/Number/Tablespoom |
| Cruciferous vegetables | 97 (52.7) | 42 (22.8) | 13 (7.1) | 22 (12.0) | 9 (4.9) | Tablespoom |
| Simple sugar | 19 (10.3) | 22 (12.0) | 24 (13.0) | 41 (22.3) | 78 (42.4) | Teaspoon/Number |
| Solid and liquid fats | 9 (4.9) | 27 (14.7) | 32 (17.4) | 64 (34.8) | 52 (28.3) | Tablespoom/Teaspoon/Number/Piece |
| Processed meats | 160 (87.0) | 15 (8.2) | 5 (2.7) | 2 (1.1) | 2 (1.1) | Cup/Grams |
| Potato chips | 70 (38.0) | 66 (35.9) | 28 (15.2) | 15 (8.2) | 5 (2.7) | Pocket |
| Carbonated drinks | 73 (39.7) | 53 (28.8) | 34 (18.5) | 17 (9.2) | 6 (3.3) | Cup |
| Tea (black) | 22 (12.0) | 7 (3.8) | 12 (6.5) | 40 (21.7) | 102 (55.4) | Cup |
| Soft drinks | 59 (32.1) | 46 (25.0) | 30 (16.3) | 37 (20.1) | 11 (6.02) | Cup |
| Olive | 133 (72.3) | 17 (9.2) | 11 (6.0) | 18 (9.8) | 5 (2.7) | Number |

**Table S2. Correlation test between different food groups**

|  | Dairy products | Meats | Fish | Nuts and seeds | Egg | Legumes | Whitebread and refined grains | Colored fruits | Other fruits | Green and orange vegetables | Cruciferous vegetables | Green leafy vegetables | Simple sugar | Solid and liquid fats | Processed meat | Potato chips | Carbonated drinks | Tea | Soft drinks | Olive |
| --- | --- | --- | --- | --- | --- | --- | --- | --- | --- | --- | --- | --- | --- | --- | --- | --- | --- | --- | --- | --- |
| Dairy products | 1.00 | 0.16^*^ | -0.11 | 0.01 | 0.16^*^ | 0.10 | 0.00 | 0.11 | 0.10 | -0.01 | -0.10 | -0.05 | 0.17^*^ | 0.09 | -0.01 | 0.00 | -0.08 | -0.05 | 0.19^**^ | -0.10 |
| Red meat | 0.16^*^ | 1.00 | -0.06 | -0.04 | -0.03 | 0.19^**^ | 0.07 | 0.18^*^ | 0.24^**^ | 0.20^**^ | 0.05 | 0.13 | 0.06 | 0.11 | 0.05 | 0.09 | 0.05 | -0.03 | 0.13 | -0.02 |
| Fish | -0.11 | -0.06 | 1.00 | 0.43^**^ | 0.04 | -0.03 | -0.05 | 0.21^**^ | 0.25^**^ | 0.39^**^ | 0.45^**^ | 0.32^**^ | -0.17^*^ | -0.10 | 0.04 | 0.05 | 0.01 | -0.02 | 0.02 | 0.43^**^ |
| Nuts and seeds | 0.00 | -0.03 | 0.43^**^ | 1.00 | 0.17^*^ | 0.17^*^ | 0.04 | 0.24^**^ | 0.19^**^ | 0.24^**^ | 0.40^**^ | 0.39^**^ | -0.06 | -0.08 | 0.04 | 0.06 | 0.05 | -0.07 | 0.04 | 0.37^**^ |
| Egg | 0.16^*^ | -0.03 | 0.04 | 0.17^*^ | 1.00 | 0.21^**^ | 0.09 | 0.01 | 0.06 | 0.18^*^ | 0.07 | 0.12 | 0.13 | 0.07 | 0.10 | 0.10 | 0.22^**^ | 0.00 | 0.12 | 0.00 |
| Legumes | 0.10 | 0.19^**^ | -0.03 | 0.17^*^ | 0.21^**^ | 1.00 | 0.13 | 0.19^**^ | 0.01 | 0.09 | 0.07 | 0.08 | 0.17^*^ | 0.16^*^ | -0.07 | 0.10 | 0.06 | -0.08 | 0.13 | 0.06 |
| White bread and refined grains | 0.00 | 0.07 | -0.05 | 0.04 | 0.09 | 0.13 | 1.00 | 0.13 | 0.08 | 0.09 | 0.05 | 0.16^*^ | 0.06 | 0.08 | -0.02 | 0.18^*^ | 0.15^*^ | 0.08 | 0.10 | 0.13 |
| Colored fruits | 0.11 | 0.18^*^ | 0.21^**^ | 0.24^**^ | 0.01 | 0.19^**^ | 0.13 | 1.00 | 0.38^**^ | 0.31^**^ | 0.20^**^ | 0.34^**^ | -0.01 | 0.08 | -0.02 | -0.01 | -0.01 | -0.05 | 0.10 | 0.23^**^ |
| Other fruits | 0.10 | 0.24^**^ | 0.26^**^ | 0.19^**^ | 0.06 | 0.00 | 0.08 | 0.38^**^ | 1.00 | 0.24^**^ | 0.21^**^ | 0.19^*^ | -0.01 | 0.02 | -0.01 | 0.04 | 0.08 | -0.06 | 0.18^*^ | 0.19^*^ |
| Green and orange vegetables | -0.01 | 0.19^**^ | 0.38^**^ | 0.23^**^ | 0.17^*^ | 0.08 | 0.09 | 0.31^**^ | 0.24^**^ | 1.00 | 0.38^**^ | 0.43^**^ | 0.01 | 0.09 | 0.08 | 0.10 | 0.07 | 0.01 | 0.04 | 0.29^**^ |
| Cruciferous vegetables | -0.10 | 0.04 | 0.45^**^ | 0.39^**^ | 0.06 | 0.06 | 0.05 | 0.20^**^ | 0.21^**^ | 0.39^**^ | 1.00 | 0.44^**^ | -0.29^**^ | -0.29^**^ | 0.09 | 0.08 | 0.09 | -0.13 | 0.03 | 0.41^**^ |
| Green leafy vegetables | -0.05 | 0.12 | 0.32^**^ | 0.38^**^ | 0.11 | 0.08 | 0.16^*^ | 0.34^**^ | 0.19^*^ | 0.43^**^ | 0.44^**^ | 1.00 | -0.05 | -0.12 | 0.03 | 0.04 | 0.08 | 0.02 | 0.12 | 0.37^**^ |
| Simple sugar | 0.17^*^ | 0.06 | -0.17^*^ | -0.05 | 0.12 | 0.17^*^ | 0.06 | -0.01 | -0.01 | 0.00 | -0.29^**^ | -0.05 | 1.00 | 0.48^**^ | -0.01 | 0.21^**^ | 0.23^**^ | 0.46^**^ | 0.25^**^ | -0.28^**^ |
| Solid and liquid fats | 0.10 | 0.11 | -0.10 | -0.08 | 0.07 | .163^*^ | 0.08 | 0.08 | 0.02 | 0.09 | -0.29^**^ | -0.12 | 0.48^**^ | 1.00 | -0.01 | 0.13 | 0.03 | 0.28^**^ | 0.15^*^ | -0.10 |
| Processed meats | -0.01 | 0.05 | 0.04 | -0.04 | 0.10 | -0.07 | -0.02 | -0.02 | -0.01 | 0.08 | 0.10 | 0.03 | -0.01 | -0.01 | 1.00 | 0.17^*^ | 0.15^*^ | 0.02 | 0.16^*^ | 0.00 |
| Potato chips | 0.00 | 0.01 | 0.05 | 0.07 | .097 | 0.10 | 0.18^*^ | -0.01 | 0.04 | 0.10 | 0.08 | 0.04 | 0.21^**^ | 0.13 | 0.17^*^ | 1.00 | 0.44^**^ | 0.19^*^ | 0.12 | -0.04 |
| Carbonated drinks | -0.08 | 0.05 | 0.01 | 0.05 | 0.22^**^ | 0.06 | 0.15^*^ | -0.01 | 0.08 | 0.07 | 0.09 | 0.08 | 0.23^**^ | 0.03 | 0.15^*^ | 0.44^**^ | 1.00 | 0.12 | 0.15^*^ | -0.01 |
| Tea (black) | -0.05 | -0.03 | -0.02 | -0.07 | 0.00 | -0.08 | 0.08 | -0.05 | -0.06 | 0.01 | -0.13 | 0.02 | 0.41^**^ | 0.29^**^ | 0.02 | 0.19^*^ | 0.12 | 1.00 | 0.09 | -0.16^*^ |
| Soft drinks | 0.19^**^ | 0.13 | 0.02 | 0.04 | 0.12 | 0.13 | 0.11 | 0.10 | 0.18^*^ | 0.04 | 0.03 | 0.12 | 0.25^**^ | 0.15^*^ | 0.16^*^ | 0.12 | 0.15^*^ | 0.08 | 1.00 | -0.03 |
| Olive | -0.11 | -0.02 | 0.43^**^ | 0.37^**^ | 0.00 | 0.06 | 0.13 | 0.23^**^ | 0.19^*^ | 0.29^**^ | 0.41^**^ | 0.37^**^ | -0.28^**^ | -0.10 | 0.00 | -0.04 | -0.01 | -0.16^*^ | -0.03 | 1.00 |
